# Case of Hemorrhagic Effusion into the Peritoneal Cavity

**Published:** 1848-12

**Authors:** Jonathan Kneeland

**Affiliations:** Borodino


ART. III.—*Case of Hemorrhagic Effusion into the Peritoneal Cavity.* By JONATHAN KNEELAND, M. D.

*To the Editor of the Buffalo Medical Journal:*

SIR:—On the night of Oct. 18th, I was summoned to the aid of John Hayne, aged 46, an Englishman, laborer on a farm. I found him in a sitting posture, groaning in a short subdued manner at each expiration; breathing somewhat hurried and thoracic; countenance expressive of great anxiety; tongue clean; and pulse 100, quick, and compressible. Several weeks previous he had been attacked with dysentery, which yielded to domestic remedies. This was followed by several attacks of colic, attended by discharges of a drop or two of blood and mucus with his fæces, for which no physician had been consulted.

The day previous to his final attack was cold and misty, the ground covered with wet snow, and during part of it he labored out of doors with his feet cold and damp, and, as he expressed it, "strained himself across the weak place in his bowels," which remained since his dysenteric attack. He retired to bed at night feeling pretty well, but about eleven o'clock awoke in great pain, and desired his wife to read prayers, declaring that he should certainly die, for "'tis a death pain, I feel it." He vomited frequently. The pain which at first was low down in the abdomen, soon spread over the whole of it, but continued most severe in the lower half. The integuments were tense; bowels moderately full, but no knotty, or tympanitic feel yet discoverable, and no evacuations *per ano.* Pressure over any part of the bowels was extremely painful; hands and feet cold; gulping and hiccough alternating. Prescribed pediluvium, sinapisms, cal. grs. v., morph. ½ gr. every hour. The vomiting subsided, but, the pain not yielding, in 3 hours I gave ½ gr. morph. and 10 drops laudanum, and repeated every half hour. No diminution of pain, nor other therapeutical effect followed, the administration of the anodyne, and at the fourth dose I desisted, as the pulse grew smaller and more frequent, and he seemed to sink rapidly. Six hours from the attack I commenced the use of enemas, emollient at first, afterwards stimulating and sedative, but dared not use the lancet, or tobacco injections, from the extreme and rapidly increasing prostration. Finally, I passed Reed's gum elastic syringe pipe into the colon, and threw up enemata several times. At first fetid gas escaped, and the enemata were soon returned without fæces. The least muscular effort now caused irregularity of the heart's action and loss of breath, and in 17 hours from the onset of pain he died.

After much delay, owing to the officiousness of a female neighbor who endeavored to prejudice the family against an autopsy, I obtained their consent, and proceeded to the examination. Upon the first incision through the peritoneal lining of the cavity of the abdomen, a dark, turbid fluid gushed out, staining the scalpel, hands, and vessel in which it was received, like blood. Nearly four quarts were collected. The contents of the abdomen were forced high up: the stomach, liver, pancreas, and spleen, being half a finger's length above the lower extremity of the sternum. These organs, as also the upper part of the ileum, the jejunum, and duodenum, were paler than usual, and the viscera in contact with the gall-bladder were not discolored from transudation of the bile, as I have often observed in other cases. The lower two feet of the ileum was *black*, not dark violet, but *black*. The caput coli was of a dull mahogany color, shading off to a light pink, and here was the only part of the bowels where minute, branching blood-vessels were discernible. The stomach was empty, but the small intestines were distended with offensive gas, and about 1½ pints of a dark tarry fluid, such as mercury sometimes causes. The colon and rectum were nearly empty, and the ileo-cœcal valve pervious; still no fluid, or gas, were found below this point. Owing to the near approach of the funeral hour, I was compelled to conclude without examining the mucous lining of the darkened intestine, as I should otherwise have done. My own impressions were, that mucous ulcerations had supervened upon the dysenteric attack, and from the conjoined influence of cold, and labor, which taxed the abdominal muscles unduly, the muscular coat and cellular tissue gave way, and thus was set up, what John Armstrong, in fasciculus 3d, page 90 to 98, calls sero-enteritis, which ran rapidly into gangrene. Abercrombie and Andral both notice a similar form of enteritis, with sanguineous effusion and blackened bowel, becoming fatal in a few hours. Abercrombie bled his fatal cases. My patient, I believe, was dying before I was called, but

I handled the lancet and his pulse alternately for some time before I fully resolved *to let him die*, without my hastening the event professionally. I expected to find intus susception, and although none was found, it seemed highly probable that a portion of the ileum had passed through the ileo-coecal valve, as here was the terminus of the black color, and above were fluid fæces, but below none whatever.

Whether the fluid was blood in a dissolved state, or sanguineous serum, let others determine. I am inclined to think the former. If so, whence came it? Does hemorrhage, even take place from serous membranes? *

Yours truly,

BORODINO, Oct. 25th, 1848. JONATHAN KNEELAND.

* NOTE, BY EDITOR.—It seems probable from the foregoing account, that ulcerations of the lower portion of the ileum existed, and that perforation of the intestine occurred at the time of the attack. The sudden access, the symptoms, and speedy termination, favor that opinion. The pathological appearances, also, go to sustain it. It would appear, under this view, that hemorrhage into the intestinal canal must have taken place shortly before perforation occurred; and the escape of blood in considerable quantity in o the canal, conjoined perhaps with some obstruction at the ileo-coecal valve, would be likely to prove the immediate cause of the perforation. The fluid contents of the peritoneal cavity, and the intestinal canal, appear to have been similar in character, making allowance for the chemical action of the intestinal gases, and secretions upon the latter.

An opportunity for a more leisured examination, (if the above supposition be correct) would have enabled the reporter of the case to have discovered the ulcerations and perforated orifice. The report would be more satisfactory, if it stated whether gas existed within the peritoneal cavity, if it had the odor of sulph. hydrogen: or if the contents of the peritoneal cavity emitted this odor; also, if the appearance of the peritoneal membrane had been more minutely described; but we are well aware, from experience, how difficult it often is, from limited time, or the opposition of friends, to conduct autopsies in private practice as ninutely as could be desired.

